# Cytology of the Blood

**Published:** 1919-08-23

**Authors:** 


### DISEASE AND THE LABORATORY.

### III. Cytology of the Blood.

AMONG the relatively simple laboratory examinations which may be of assistance to the physician not only for diagnostic purposes but also in some instances prognostically, comes the quantitative and qualitative examination of the cellular contents of the blood stream. Yet one believes that in practice not a tenth part of the possible indications to be derived from these simple estimations are made use of by a great number of practitioners. It is not to be expected that such work can be regularly applied to the average surgery case, and it is to the obscure case, the case with apparently mild initial symptoms but which obstinately refuses to improve, that we more particularly refer. In the following notes it is attempted simply to outline a few of the situations in which a reading of the blood picture may be more especially valuable. It is impossible here to give detailed descriptions of apparatus, much of which is now of more historical than practical interest, and after brief mention of one method, which is probably the best for each particular estimation, we will proceed to a few blood pictures which are typical of certain clinical conditions of particular interest at the present stage of medical affairs.

### ENUMERATION OF CELLS.

To enumerate both red and white cells, Burker's hæmocytometer is by far the best. It has many advantages over the old Thoma-Zeiss form. In the first place there are formed at once and from the same drop of blood two separate counting chambers, the one controlling the other. Also both the red and white cells can be counted on the same drop of blood, while the whole system is considerably quicker and easier, losing, however, nothing in the accuracy of the determination. To estimate the percentage of hæmoglobin use is still advisedly made of Haldane's hæmoglobinometer, while in making films for more individual examinations of the cells the best way is to take two clean glass slides, catch a drop of blood upon the end edge of one of them, at once squarely apply this edge to the upper surface of the second slide. The blood runs along this edge and the upper slide is drawn firmly but with a slightly jerking movement along the whole length of the lower, producing a complete film of alternating thin and thick bands of cells. In the cases of leucocytosis and leucopenia the bands are found correspondingly useful, but if film is particularly for red cell, or more particularly for blood parasite, examination then it is essential to make the whole film as thin and evenly distributed as possible. There is not much to choose between Leishmann's and Jenner's stains. In practice, the former is undoubtedly preferable for parasites, while the latter, which can be kept in a wide-mouthed stoppered vessel, allowing complete immersion of the slides, is probably best for routine work.

### THE NORMAL BLOOD.

For detailed description of the commonly encountered blood cells the reader may refer to any good standard text-book, and the common average estimations are too well known to call for mention here, but a few points regarding pseudo-abnomalities are perhaps worth noting. It is not uncommon to find a red cell count of well over 5 million per cm. with a colour index of only .9 or less in a perfectly healthy male, while under similar good health a woman may show less than 4 million reds with an even lower colour index. In infants under 12 months, the number of red cells is often very high, up to even 6 million per cm. But the greater variations occur in the white count of normal people, 8,000 or even higher being quite often met in health, while the so-called digestion leucocytosis is very real and oft-encountered. During menstruation also normal changes occur, both red cells and hæmoglobin being reduced and a slight increase in the whites occurring. Finally, a number of instances of leucoid lymphocytosis may be noted in the latter half of pregnancy disappearing completely after uncomplicated parturition.

### DIAGNOSIS OF DISEASE.

According to Price-Jones in his compact little volume entitled "Blood Pictures," from which we take some of the examples given, broadly speaking, a leucoid type of blood is associated with a coccal infection, a lymphoid type with a bacillary infection, and a lymphoid plus large mononuclear type with a protozoal infection. Mixed infections, late or superimposed infections, &c., may all produce misleading blood counts, and diagnosis from a blood picture should not be made without due guidance from clinical evidence, history, and any intercurrent complications. Another most important point is that although a single blood examination may be of great value and may even provide a key to diagnosis, yet it cannot be too strongly impressed that really satisfactory conclusions can be derived only if repeated complete blood examinations are made. Taking first the different types of bacterial infection, we may on the strength of a great number of counts generalise by saying that with a leucocytosis of 10,500 per cm. or over, and the polymorphonuclears numbering 75 per cent., and apart from the corresponding decrease of lymphoid cells, no other abnormalities presented, it is almost certain that we are dealing with an acute coccal infection. If the polymorphs rise to about 90 per cent. there is the greatest probability that suppuration has occurred.

### INFECTION BY BACILLI.

Here we may include the tubercle, coli-typhoid, diphtheroid groups, &c., and it is found that if inflammation be due to a pure infection by one of these, then the blood picture is of the lymphoid type.

Previous articles appeared on July 26 and August 9, p. 471.

**Disease and the Laboratory**—(*continued*).

There may not be an actual leucocytosis at all, in fact, the white count is often reduced, but the small and large lymphocytes are relatively greatly increased at the expense of the polymorphonuclears. In practice, so frequently does secondary coccal infection occur in these cases, that the above appearance becomes rapidly masked, a typical coccal count gradually superseding the lymphoid type. The point is that in early uncomplicated stages considerable information may be provided by the lymphoid predominance, while the permanent or periodic masking by the leucoid type may give useful indications of the progress of a case. But as already mentioned, the full value is only obtained if repeated blood examinations be made. Let us take the typhoid fever as a typical example with its early leucopenia of lymphoid type, which changes to leucoid and leucocytosis as complications arise. In uncomplicated cases the leucopenia will persist unchanged into the second and third weeks of the disease, and meanwhile departure from the type is at least an early sign for precaution on the part of the M.O. in charge.

Of the other bacillary infections, uncomplicated diphtheria has a lymphoid picture, while mixed or pure coccal tonsillitis gives the opposite result, so that a rapid diagnosis may sometimes be made without waiting for 24 hr. culture. Here also at the start, polycythæmia may be present, followed later by a chlorotic type of anæmia with polychromasia, &c. Influenza provides an interesting example in that pure Pfeiffer infection produces a lymphoid count which is frequently masked as secondary lung invasions begin to occur.

### Protozoal Infections.

According to recent records on these infections the blood picture is usually fairly definite. As in malaria and syphilis, there is usually some oligocythæmia and lowered Hb percentage, together with regenerative and destructive red cell signs. A certain diagnosis is of course obtained by the detection of the parasite itself, but in the event of failure to find this, many authorities agree that there is a lymphoid blood picture with a considerable relative and absolute increase of the large mononuclear cells. To many a large monoculear leucocytosis is pathognomonic of uncomplicated protozoal disease. It is unnecessary here to recount the details of the commoner types of true blood disease, for their main characters are found in every text-book and taught to every student, but one may advantageously point out one or two considerations in connection with the films from a person suffering from malignant disease.

### Malignancy.

In the case of early carcinoma before development of the typical cachexia and metastases the usual picture is stated to be a low degree of a large mononuclear and polymorphonuclear leucocytosis. Later red cells are diminished and a chlorotic anæmia sometimes supervenes, but on the whole very little guidance can be obtained from these facts One or two useful instances occur as follows. Price-Jones notes that in cases of gastric carcinoma, when the polymorphonuclear leucocytes have progressively increased in number, metastases in the liver are probably present. Again, a clinical suspicion of growth may often be negatived by a high leucocytosis of the suppurative type. In gastric cases decision between malignant and simple ulcerative types may be helped by the absence of the malignant picture and presence of a simple secondary anæmia. A number of abdominal conditions are simulated by vagaries of so-called intestinal stasis, which gives by itself a typical lymphoid picture as opposed to the leucocoid type commonly produced by complications with secondary infections or even malignancy. One gives these examples, not as decisive in themselves, but as illustrating the possibilities of diagnostic value in blood examinations. Much of the available information is regrettably indefinite in all except the true intrinsic blood diseases, and brief and incomplete as these notes are, it is hoped they may at least indicate that there are many openings for more fruitful research in this particular branch of laboratory work.

